# CeRA-eSP: Code-Expanded Random Access to Enhance Success Probability of Massive MTC

**DOI:** 10.3390/s22207959

**Published:** 2022-10-19

**Authors:** Jiseung Youn, Joohan Park, Joohyun Oh, Soohyeong Kim, Seyoung Ahn, Sunghyun Cho, Sangwoo Park, Cheolwoo You

**Affiliations:** 1 Department of Applied Artificial Intelligence, Hanyang University, Ansan 15588, Korea; 2 Department of Computer Science and Engineering, Hanyang University, Ansan 15588, Korea; 3 Department of Information & Communications Engineering, Myongji University, Yongin 17058, Korea

**Keywords:** random access, code-expanded random access, massive machine-type communications, Internet of Things

## Abstract

With the growing interest in the Internet of Things (IoT), research on massive machine-type communication (mMTC) services is being actively promoted. Because mMTC services are required to serve a large number of devices simultaneously, a lack of resources during initial access can be a significant problem when providing mMTC services in cellular networks. Various studies on efficient preamble transmission have been conducted to solve the random access problem of mMTC services. However, supporting a large number of devices simultaneously with limited resources is a challenging problem. In this study, we investigate code-expanded random access (CeRA), which extends the limited preamble resources to the code domain to decrease the high collision rate. To solve the existing CeRA phantom codeword and physical uplink shared channel (PUSCH) resource shortage problems, we propose an optimal preamble codeword set selection algorithm based on mathematical analysis. The simulation results indicate that the proposed code-expanded random access scheme to enhance success probability (CeRA-eSP) achieves a higher random access success rate with a lower access delay compared to the existing random access schemes.

## 1. Introduction

With the emergence of 5G services, such as smart cities, smart grids, and smart homes/buildings, interest in Internet of Things (IoT) networks has increased significantly [1,2,3]. To ensure the connectivity of large-scale networks, the international telecommunication union radiocommunication sector (ITU-R) defined massive machine-type communications (mMTC) as one of the main use cases of 5G [4,5]. The main features of mMTC are high connectivity of up to 1 million devices per square kilometer, small data volume, sporadicity, and delay tolerance [1,6]. Because the characteristics of mMTC services are different to those of existing human-type communication (HTC)-based services, an mMTC-specialized study is required.

A representative problem of mMTC is the preamble collision that occurs during the random access (RA) process. Preamble collisions occur when devices transmit the same preamble sequence during RA. In mMTC, massive devices transmit preamble sequences simultaneously, resulting in a higher collision rate in the preamble transmission phase. The access delay increases because most devices retransmit the preamble owing to the high collision rate. A high collision rate causes a decrease in the RA success rate. Therefore, the high collision rate problem is considered a representative problem of mMTC services [7,8].

Extending the preamble resource is one approach to solving the high collision rate problem. Among the preamble extension schemes, a code-expanded random access (CeRA) scheme was proposed. The CeRA scheme operates based on a preamble codeword. A preamble codeword refers to a longer preamble format composed of serial preamble sequences transmitted in consecutive RA slots. We assumed that the number of preamble sequences that can be transmitted in each random access occasion (RAO) is *M* and the number of preamble sequences constituting one codeword is $L_{c}$. If the base station (BS) can recognize the preamble sequences for each RAO, the number of preamble codeword combinations is $M^{L}$. The CeRA scheme is an innovative method that can exponentially increase preamble resources, considering that existing preamble extension methods linearly increase preamble resources [9,10]. Because the preamble collision rate is significantly reduced as the number of selectable preamble codewords increases, the CeRA scheme can efficiently solve the high collision-rate problem.

Furthermore, the CeRA scheme does not require significant changes in the preamble transmission and detection processes. To discuss the difference between the CeRA scheme and the existing RA scheme, it is necessary to know the preamble codeword transmission and identification processes. The preamble sequence transmitted in successive RA slots should be concatenated as long as the codeword length for the BS is able to successfully identify the preamble codeword. The CeRA scheme uses a virtual frame composed of consecutive RA slots for preamble codeword detection. The BS records the successfully transmitted preamble sequences in each RA slot that are included in the virtual frame. After the preamble transmission in the virtual frame is completed, the BS deduces the possible preamble codeword combinations based on the preamble sequence observations recorded for each RA slot. It can be observed that the preamble codeword reception process of the BS does not require any special change other than the transmission in units of virtual frames. With respect to the preamble transmitter, the devices transmit a random preamble sequence in the same manner as the conventional method. Because the receiver and transmitter do not require significant changes in the RA process, the CeRA scheme allows for the accommodation of the bursty RA load of the mMTC with minimal changes in the MAC layer [9].

Despite its various advantages, the CeRA scheme has unresolved problems. A representative problem is the codeword ambiguity problem that occurs during the deduction process of the preamble codeword. In a conventional RA scheme, the BS can accurately discern the actual transmitted preamble sequence for each RA slot. By contrast, the CeRA method deduces the preamble codeword based on the preamble observation for each random access slot, allowing it to recognize the preamble codewords that have not been transmitted. For example, we consider a situation in which *n* devices transmit a preamble codeword of length $l_{c}$. If the devices transmit different preamble sequences in all RA slots, the actual number of preamble codewords transmitted by the devices is equal to the number of devices *n*. However, the BS deduces the number of $n^{l}$ possible combinations based on the preamble sequence observations for each RA slot. A preamble codeword that the device did not actually transmit was deduced by the BS, which is defined as a phantom codeword. The codeword ambiguity problem refers to a problem in which it becomes difficult to identify the actual preamble codeword as the proportion of phantom codewords increases.

The codeword ambiguity problem causes a shortage of PUSCH resources. In the legacy RA scheme, the BS recognizes the transmitted preamble sequences and sends a random access response (RAR) message to the user-sent preamble sequences. The device allocates a dedicated PUSCH resource using an RAR message when the preamble transmission is successful. The PUSCH resource is a limited uplink resource for the device to transmit data packets and control signals after the preamble transmission. The PUSCH shortage problem occurs when PUSCH resources are wasted on phantom codewords. Specifically, the majority of deduced preamble codewords are phantom codewords when codeword ambiguity is high. If the BS randomly allocates PUSCH resources, most of them are allocated to the phantom codeword. Because the phantom codeword is not a preamble codeword transmitted by an actual device, this results in a waste of PUSCH resources. If PUSCH resources are wasted, the PUSCH resources that need to be allocated to the transmitted preamble codewords become insufficient. Because devices that are not allocated PUSCH resources are dropped during the RA process, the shortage of PUSCH resources is directly related to the degradation of the RA performance.

As mentioned previously, two main factors contribute to the degradation of the RA performance: preamble collision and codeword ambiguity. If the BS attempts to solve the high collision rate by using a large-scale preamble codeword set, the codeword ambiguity problem occurs because of a non-overlapping preamble transmission, which incurs a large, deduced preamble set. By contrast, if the BS utilizes a small preamble codeword set to solve the codeword ambiguity problem, the performance of the RA deteriorates owing to the preamble collision. Because both factors are closely related to performance according to the size of the preamble codeword set, preamble collision and codeword ambiguity have a trade-off relationship. To maximize the RA performance, CeRA studies that consider both factors should be conducted.

In this paper, we propose a code-expanded random access to enhance success probability (CeRA-eSP) scheme that considers preamble collision and codeword ambiguity to increase the RA success rate and PUSCH resource utilization. The proposed scheme consists of a preamble codeword-set selection phase and PUSCH resource allocation phase. In the preamble codeword set selection algorithm, we design three probabilistic analysis models with respect to the preamble transmission success rate, preamble codeword utilization rate, and PUSCH timeout rate. Because the three factors have a trade-off relationship, we analyze the random-access success rate by considering these three factors and select the preamble codeword set that maximizes the random access success rate. In the PUSCH resource allocation phase, we propose an improved PUSCH allocation procedure that utilizes the PUSCH waiting message. The proposed PUSCH allocation scheme can improve PUSCH resource utilization by using unused PUSCH resources at a lower access intensity. Finally, we validate the improvement of the proposed CeRA-eSP using a performance analysis simulation. The simulation results show that the proposed CeRA-eSP achieves the highest random-access success rate while maintaining the lowest access delay.

In summary, the main contributions of this study are as follows:

- We propose a CeRA-eSP scheme to improve the RA success rate and PUSCH resource utilization. The proposed CeRA-eSP scheme consists of two phases. One is the preamble codeword set selection phase that devices can select in each RAO. The other is the PUSCH resource allocation phase to the preamble codeword recognized by the BS.
- We propose a preamble codeword set selection algorithm based on the number of active devices to maximize the RA success rate. The proposed preamble codeword set selection algorithm consists of three kinds of analytic models for the preamble transmission success rate, preamble codeword utilization rate, and PUSCH timeout rate.
- We propose an improved PUSCH allocation scheme based on the PUSCH waiting message to improve the utilization of PUSCH resources.
- We show the performance of the proposed CeRA-eSP scheme in terms of the access delay, preamble collision rate, and RA success rate. The proposed CeRA-eSP scheme has the lowest access delay among the benchmark schemes and shows the highest RA success rate.

The remainder of this paper is organized as follows. Related studies on contention-based RA, CeRA, and PUSCH resource allocation are introduced in Section 2. The system model for the preamble codeword-based RA is introduced in Section 3. Analytical models for a successful preamble transmission rate, preamble ambiguity, PUSCH resource limitation, and preamble codeword set allocation scheme are introduced in Section 4. The experimental results and performance evaluation are presented in Section 5. Finally, Section 6 concludes the paper.

## 2. Related Works

### 2.1. The Legacy Contention-Based Random Access Procedure

The contention-based RA procedure is a reference method used in the existing RA scheme and it has difficulty supporting massive connectivity. Therefore, a number of studies on contention-based RA considering the massive RA of 5G have been conducted [11,12,13,14,15,16,17,18,19,20,21,22,23,24].

A study was conducted on the access class barring method proposed by the 3GPP standard organization to solve the high collision problem of mMTC. The authors of [11] designed a random access channel (RACH) success probability analysis model for the three coverage enhancement groups based on the 3GPP NB-IoT standard. The author suggested that with the existing access class barring (ACB) and back-off (BO) algorithms, the RA success rate can be approximately doubled through RA management for the three coverage enhancement groups compared to the massive RA situation in a single group. The authors of [12] analyzed the performance of power ramping, back-off, and access class barring (ACB) algorithms to solve the congestion situation of mMTC. Studies on the ACBs utilized in the 3GPP NB-IoT standard were conducted in [13]. The authors improved the collision rate by proposing an ACB configuration method that selects the optimal barring parameters in real time. In [14], joint optimization of the preamble selection and access barring methods was performed. The author derived a solution to the average RA throughput maximization problem using the block coordinate descent method, which is a non-convex problem.

Numerous studies have been conducted on increasing the service satisfaction rate by allocating prioritized preambles according to the requirements of each device. The authors of [15] proposed a distributed queuing-based content resolution scheme for MAC layer load estimation. Through the design of the distributed queue-based framework, the author solved the congestion of massive access and guaranteed the priority of device access. In [16], various device access requirements were considered. Using the proposed access priority provisioning technology, devices can satisfy the delay requirement by transmitting multiple preambles according to the priority order. The authors of [17] studied a method for distinguishing devices in the access control process using a tagged preamble. The authors of [17] studied a method for distinguishing devices in the access control process using a tagged preamble. Through joint optimization of the tagged preamble and access-class barring parameters, the author improved the RA success rate. In [18], the MAC and PHY layer enhancement of RA was studied to consider the QoS requirements of the various service types of mMTC.

To improve the RA performance in mMTC, a machine-learning-based approach was studied. The authors of [19] proposed a reinforcement learning (RL)-based ACB method that can adaptively select the barring rate. The proposed scheme improved the access success probability through RL-based dynamic barring factor selection. The authors of [20] studied the ACB method by considering the energy consumption and delay constraints of the device. In this study, a deep reinforcement learning approach was used to obtain the real-time mean barring time and barring rate. To resolve preamble collisions, [21] proposed a preamble selection method based on multi-agent reinforcement learning. In addition to the aforementioned studies, various other approaches to mMTC have been studied. In [22], a method for utilizing unused PUSCH resources was proposed to support devices that failed RA in mMTC. The proposed PUSCH resource reallocation algorithm increased the RA success rate at the cost of energy consumption. To support a large number of connections in mMTC, the authors of [23] used a compressive sensing method. The authors reduced the RA collision rate by utilizing the sporadicity characteristics and a non-orthogonal pilot. The authors of [24] proposed a novel grant-free RA method based on non-orthogonal multiple access (NOMA) to fulfill the massive connection requirements of 5G mMTC. The proposed method achieved a higher RA throughput than the NOMA-based RA method in various user-density environments.

However, in previous studies on contention-based RA, it was difficult to reduce the collision rate owing to the lack of reference preamble sequences. To support massive connectivity, it is necessary to consider the preamble extension.

### 2.2. Code-Expanded Random Access Procedure

To utilize the preamble extension in an mMTC environment, various studies related to CeRA have been conducted [9,10,25,26]. In [10], the RA procedure and frame structure of CeRA were designed. The authors proposed an adaptive CeRA method to reduce the number of phantom codewords and improve the utilization of preamble codewords by adaptively allocating them according to the user device density. The authors of [25] allowed devices to transmit preamble codewords that remove the similarity between codewords through *q*-ary maximum average distance code to solve the problem of CeRA’s low preamble codeword utilization. The author of [25] proposed a codeword similarity removal scheme based on *q*-ary maximum average distance code to solve the low-preamble codeword utilization problem of CeRA. In [9], codeword ambiguity and PUSCH resource allocation probabilities were analyzed. To improve the RA success probability, a balanced preamble codeword set allocation scheme with low ambiguity and high PUSCH resource allocation probability was proposed. The authors of [26] conducted a study to increase the success rate of grant-free RA through successive preamble transmissions. The author proposed a massive MIMO-based user equipment detection algorithm to improve the RA success rate based on preamble codeword transmissions.

Previous studies have analyzed the preamble codeword utilization in the preamble transmission process. Most studies do not consider the utilization of PUSCH resources, and it is possible that the RA success rate will decrease owing to a shortage of PUSCH resources. In the case of studies considering PUSCH, it is difficult to allocate an appropriate preamble codeword set because the preamble codeword increases exponentially in units of the codeword length. To improve the overall RA success rate, it is necessary to consider the efficiency of PUSCH resource allocation and preamble codeword set allocation.

## 3. System Model

### 3.1. Target System Model

**Figure 1:**
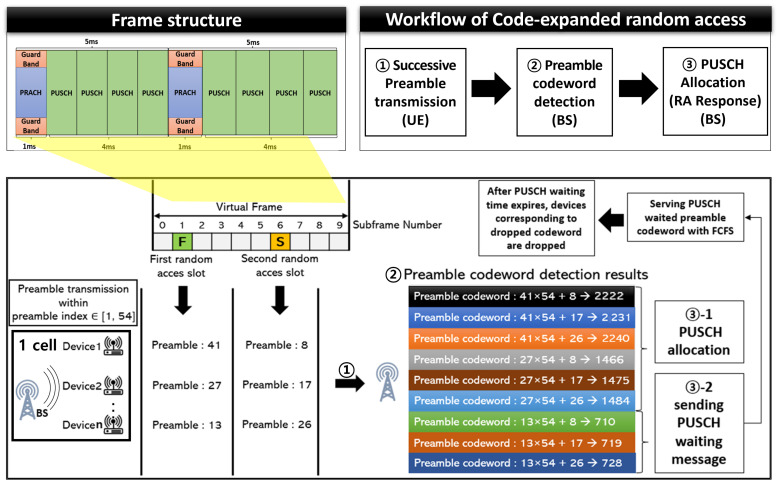
System model.

We considered a single cell consisting of *N* mMTC devices and one BS. The overview of the target system model is illustrated in Figure 1. A device becomes active when data are available for transmission. The activation patterns of devices are sporadic because the data collection of the devices differs depending on the sensing environment. This sporadicity renders it difficult to predict when and which device will be active in a single RA slot. The number of activated users at time $t\in (0,T_{A})$ follows the beta distribution. Specifically, the beta distribution is used with the parameters α=3 and β=4 of the 3GPP standard as follows [27]:

(1) $$p\left(t\right)=\frac{t^{\alpha -1}{\left(T-t\right)}^{\beta -1}}{T_{A}^{\alpha +\beta -1}B\left(\alpha ,\beta \right)},$$

where B(α,β) denotes the beta function, $\int_{1}^{0}t^{\alpha -1}{\left(1-t\right)}^{\beta -1}dt$. The beta distribution is commonly used in mMTC RA scenarios where bursty traffic occurs [24,28].

As shown in Figure 1, the PUSCH waiting message was used in our system model. The PUSCH waiting message is used when the PUSCH resources are insufficient to support all the preamble codewords that are transmitted in the specific RAO. In this case, some preamble codewords cannot be allocated using PUSCH resources because of the lack of PUSCH resources in the corresponding RAO. Devices that have transmitted the PUSCH resource-unallocated preamble codeword are dropped in the existing RA scheme, leading to a decrease in the RA success rate. To prevent a decrease in the RA success rate, the BS identifies the successfully received preamble codeword and broadcasts the PUSCH waiting message to the PUSCH resource-unallocated devices. The devices that correspond to the waiting preamble codeword do not perform RA retransmission in the next RAO, although they monitor the RAR message for the given PUSCH waiting time window.

Among the existing RA schemes, the back-off scheme improves the RA success rate by distributing the access intensity to one RAO [29]. Similar to the back-off scheme, the PUSCH waiting message can improve the RA success rate by distributing high-access requests over other RAOs. Specifically, in a situation where high access intensity continues, a large preamble codeword is set to lower the high collision rate. If the size of the transferable preamble codeword set is larger than that of the PUSCH resource, the BS transmits a PUSCH waiting message to some devices because of the lack of PUSCH resources. Devices that have received the PUSCH waiting message wait for the PUSCH waiting expiration and retransmit the preamble codeword. Therefore, the PUSCH waiting message can lower the access intensity by setting a time interval for devices with high access intensity.

**Figure 2:**
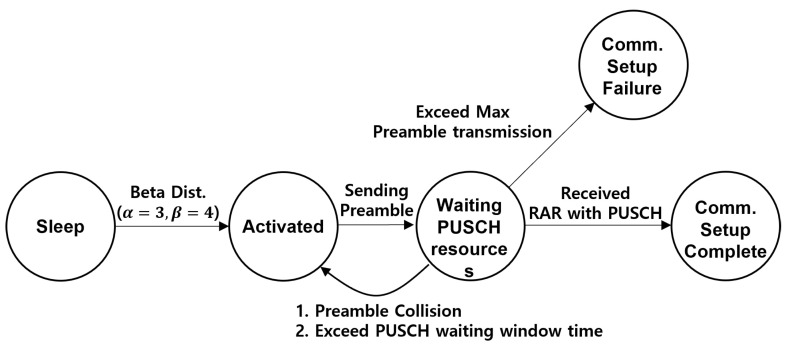
State transition diagram of an IoT device.

Figure 2 shows the state transition of the device during the RA. The active device had sufficient power and data required for RA, and the activation of the device indicated that the device could perform RA. After the preamble transmission, the device waits for the RAR message including the PUSCH resource. If the PUSCH resource is allocated, the device transitions to the complete state of the communication setup; otherwise, the device performs RA retrial up to the maximum number of preamble transmissions allowed. When the devices reach the maximum preamble transmission, they transition to the communication setup failure state. Our system model contained a single-shot RA case for simplicity of the analysis model. Single-shot RA means that once a device falls into a communication setup failure state, the RA reattempt for the device is no longer considered. Notably, the system model consisted of the RA retrial until it reached the maximum number of preamble transmissions without further retransmission after the communication setup failure.

### 3.2. Frame Structure and the Legacy Uplink Radio Resources

**Figure 3:**
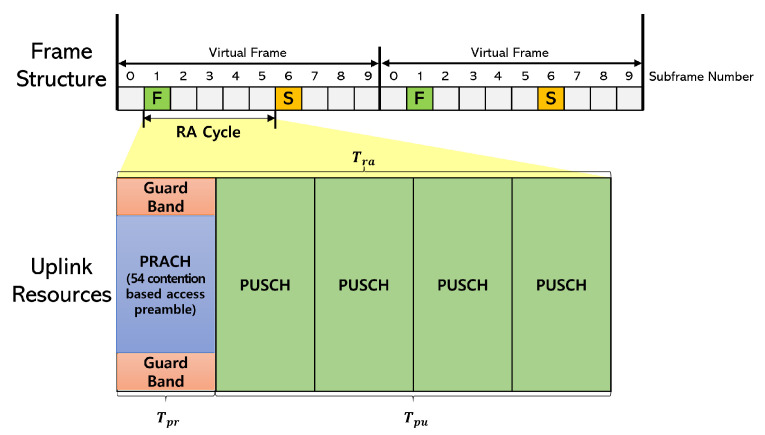
Frame structure of codeword-based random access (codeword length $L_{c}=2$ and PRACH configuration = 6).

Figure 3 shows the frame structure and uplink radio resources of CeRA. The specified subframe according to the PRACH configuration is utilized as an RA slot for the preamble transmission. The RA slots of codeword length $L_{c}$ form one virtual frame for the successive transmission of the preamble sequence. Devices randomly transmit one of the *M* preamble sequences in each RA slot, similar to the legacy RA scheme. Because a virtual frame consists of $L_{c}$ RA slots, the devices transmit a total of $L_{c}$ preamble sequences during one virtual frame duration. After transmission of the preamble codeword, the BS deduces the preamble codewords based on the preamble sequence observation in each RA slot. A preamble sequence observation refers to a process in which the BS confirms the results of the preamble sequences that are successfully received in each RAO. The BS deduces the preamble codewords by combining the preamble sequences observed in the virtual frame.

The RA cycle defined in [30] was used in the system model to consider uplink radio resources for RA. The RA cycle has a length of $T_{ra}$ ms and consists of a PRACH resource of $T_{pr}$ ms and a PUSCH resource of $T_{pu}=T_{ra}-T_{pr}$ ms. In the 5G IoT random access scenario, the RA cycle duration and PRACH duration are commonly set to $T_{ra}=5$ ms and $T_{pr}=1$ ms [5,21]. The PRACH resource consists of 839 subcarriers at 1.25 kHz. Because the PRACH resource includes *M* orthogonal contention-based preamble sequences, the devices use the PRACH resource for the preamble transmission. The PUSCH resource consists of 72 subcarriers at 15 kHz. The PUSCH resource is used by devices to transmit a connection request and data after transmitting the preamble. The maximum number of available transmissions in PUSCH, as described in [30], can be calculated as follows:

(2) $$N_{ST}=\left\lfloor \frac{L_{RAC}}{\left\lceil \frac{\Theta_{max}}{rlog_{2}\left(I\right)}\right\rceil }\right\rfloor \cdot T_{pu},$$

where $L_{RAC}=6048$ denotes the number of selectable layers supported by sparse code multiple access in one RAO, $\theta_{max}=160$ bits denotes the data transmission size for each device, r=1 denotes the code rate, and I=4 denotes the number of constellation points. The above-mentioned values are calculated according to the formula in [30] and follow the network standard of [27]. For the special case where the codeword length $L_{c}=2$, as shown in Figure 3, the total number of subcarriers is $T_{ra}=10$ and the number of PRACH subcarriers is $T_{pr}=2$; thus, the number of PUSCH subcarriers is $T_{pu}=8$. When $N_{ST}$ is the maximum number of available transmissions in the PUSCH, it can support as many devices as $N_{ST}=144$ using Equation (1). If the number of deduced preamble codewords exceeds the allocable PUSCH resources, the BS selects random preamble codewords up to $N_{ST}$ to send RAR messages and sends PUSCH waiting messages to extra preamble codewords.

## 4. Proposed Scheme

**Figure 4:**
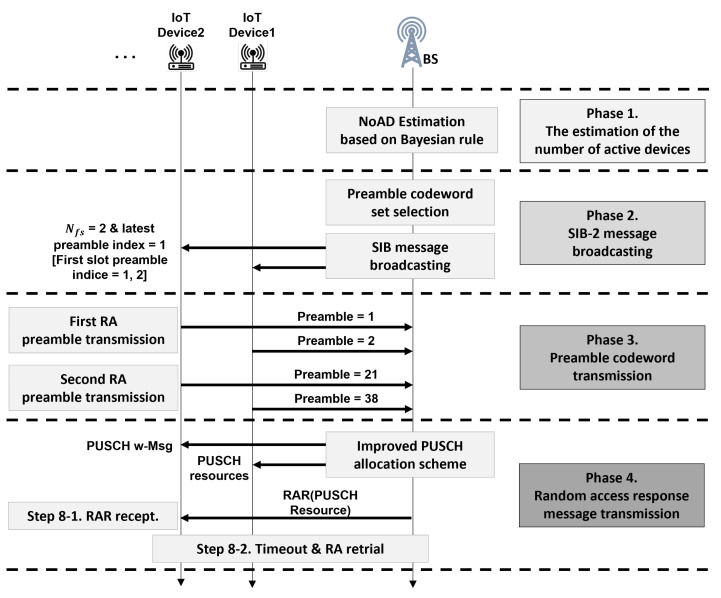
Overall procedures of the proposed CeRA-eSP scheme.

In this section, we describe the proposed code-expanded random access to enhance the success probability (CeRA-eSP). The CeRA-eSP is a random access scheme to reduce the preamble collision rate in the mMTC environment and improve the actual random access success rate while considering uplink resource limitations. The overview of the proposed CeRA-eSP is shown in Figure 4. The CeRA-eSP consists of four phases: estimation of the number of active devices, SIB-2 broadcasting, preamble transmission, and RAR response.

First, the number of active devices estimation phase aims to estimate the current access intensity indicator, which is utilized in the preamble codeword set selection algorithm. We use the Bayesian rule-based estimation scheme proposed in [28] to measure the number of active devices in each RAO. The Bayesian estimation scheme requires an a priori distribution and the number of idle preambles in the previous RAO to estimate the posterior distribution of the current RAO. Because the posterior distribution estimated at time slot *t* is used as an a priori distribution in the next time slot t+1, it is possible to obtain the number of active devices in the current RAO using a recursive Bayesian estimation. The number of active devices for all RAOs is calculated using the following recursive calculation based on the Bayesian rule:

(3) $$\nu \leftarrow \nu \left(1-\frac{I}{M}\right){\left(1-e^{-\frac{\nu }{M}}\right)}^{-1},$$

where *I* is the number of unselected preambles and ν is the mean value of the activated device.

Second, the SIB-2 message broadcasting phase is a process for selecting the optimal preamble codeword set to optimize random access performance. In a legacy CeRA scheme, an oversized preamble codeword set, which increases exponentially as the codeword length increases, causes the codeword ambiguity problem mentioned in Section 1. We refine the scale of the codeword set by limiting the transferable preamble sequences in the first RA to solve the codeword ambiguity problem. Due to the trade-off relationship between codeword utilization and the collision rate, it is necessary to select the optimal preamble codeword set considering both factors in order to improve the overall RA success rate. We design mathematical models for three key RA performance indicators, (1) preamble transmission success rate, (2) preamble codeword utilization rate, and (3) PUSCH timeout rate, which are described in Section 4.1, to implement the preamble codeword selection algorithm. The BS determines the number of preamble sequences that can be transmitted in the first random access slot for the current RA cycle according to a mathematical model based on the estimation of the number of active devices. The BS sequentially allocates the preamble sequences of the first random access slot and continuously records the last number of the preamble indexes of the first RA slot that were used in the previous RA cycle. Through the last preamble index and preamble sequence scale information, the BS broadcasts the available preamble sequence indexes of the first random access slot in the current RA cycle using the SIB-2 message. The devices can identify the transferable preamble codeword set in the current RA cycle based on the SIB-2 message information.

Third, the preamble codeword transmission phase aims to reduce the collision rate in the preamble transmission phase by transmitting a large-scale preamble codeword. In Figure 4, the devices randomly transmit one of the transferable preamble sequences in the first RA slot. Thereafter, the devices randomly transmit one of the *M* preamble sequences in the second RA slot. Through two consecutive preamble sequences, the device can transmit one preamble codeword. Devices that have transmitted the preamble codeword monitor the signal on the physical downlink shared channel for a predefined RAR receive timer, which is used in legacy random access procedures.

Finally, the RAR message transmission phase is a process for allocating uplink resources to devices that have successfully transmitted the preamble codeword. In legacy RA, low PUSCH utilization decreases the RA success rate as the number of PUSCH non-allocable devices increases. In order to improve the PUSCH resource utilization, we propose an improved PUSCH allocation procedure based on the PUSCH waiting message. The PUSCH waiting message is transmitted to devices that have not been allocated PUSCH resources when the allocable PUSCH resource is less than the number of received preamble codewords. Since the proposed PUSCH allocation method does not require significant changes in the RAR transmission process, we can follow the legacy random access procedure with small modifications.

Among the four phases of the proposed CeRA-eSP scheme mentioned above, phases 1 and 3 are the same as in the procedure of the legacy CeRA. As the main difference from the legacy CeRA, first, we propose the preamble codeword set selection algorithm to improve the RA success rate in phase 2 of the CeRA-eSP. Second, we propose an improved PUSCH allocation scheme based on a novel PUSCH waiting message to improve PUSCH utilization. In the following subsections, we describe the two proposed schemes in phases 2 and 4.

### 4.1. Preamble Codeword Set Selection Algorithm

In order to select an optimal preamble codeword set to improve the RA success rate, it is necessary to consider all the RA factors. As mentioned in Section 1, there is a trade-off relationship between the collision rate, which is also called the preamble success rate, and the codeword ambiguity. In addition, the device needs to be allocated a dedicated PUSCH resource in order to succeed in RA; it is also necessary to consider the degradation of RA performance due to PUSCH resource limitations. To consider all of the RA factors, we first design the mathematical analysis model of the preamble transmission success rate, preamble codeword utilization rate, and PUSCH timeout rate analysis. The first two metrics are related to the trade-off between the collision rate and codeword ambiguity, and the last metric is used to calculate the PUSCH non-allocable preamble codeword rate. After the analysis model description, we describe the proposed algorithm to select the optimal preamble codeword set considering the three metrics mentioned above.

#### 4.1.1. Preamble Transmission Success Rate Analysis Model

Given the number of active devices, the success rate for the average number of successful preambles is proposed in [28] as follows:

(4) $$S=\frac{\nu e^{-\frac{\nu }{M_{c}}}}{M_{c}},$$

where S is the success probability of the preamble transmission and $M_{c}$ is the number of preamble codewords.

#### 4.1.2. Preamble Codeword Utilization Rate Analysis Model

We define $N_{fs}$ as being the number of transferable preamble sequences that are broadcasted by the BS in the first RA slot, and $N_{chosen}$ as being the number of actually transmitted preamble sequences by devices in the first RA slot. A device transmits a random preamble sequence among the $N_{fs}$ preamble sequences. Therefore, $N_{chosen}$ can be calculated as follows:

(5) $$N_{chosen}=N_{fp}\left(1-{\left(1-\frac{1}{N_{fp}}\right)}^{\nu }\right).$$

In (5), the value ν, which means the number of active devices in the current RA cycle, is a large value in mMTC. We assume that $N_{fs}≃N_{chosen}$ since ${\left(1-\frac{1}{N_{fp}}\right)}^{\nu }$ has a value close to zero. According to the above assumption, the probability that a specific preamble codeword is chosen by at least one device is calculated as follows:

(6) $$P_{chosen}=1-{\left(1-\frac{1}{MN_{fp}}\right)}^{\nu }.$$

The expected number of codewords chosen by at least one device can be calculated as follows:

(7) $$E\left[N_{codeword}\right]=P_{chosen}\cdot M\cdot N_{fp}=M\cdot N_{fp}\left(1-{\left(1-\frac{1}{MN_{fp}}\right)}^{\nu }\right).$$

In a specific situation where the preamble codeword length $L_{c}=2$, the expected number of preamble codewords selected in the first and second RA slots can be calculated as follows:

(8) $$E\left[N_{fp}\right]=N_{fp}\left(1-{\left(1-\frac{1}{N_{fp}}\right)}^{\nu }\right)$$

(9) $$E\left[N_{sp}\right]=M\left(1-{\left(1-\frac{1}{M}\right)}^{\nu }\right).$$

The average number of preamble codewords deduced by the BS is calculated as follows:

(10) $$E\left[N_{de}\right]=E\left[N_{fp}\right]\times E\left[N_{sp}\right]=M\cdot N_{fp}\left(1-{\left(1-\frac{1}{M}\right)}^{\nu }\right)\left(1-{\left(1-\frac{1}{N_{fp}}\right)}^{\nu }\right).$$

As the preamble codeword utilization refers to the ratio of the actually transmitted preamble codewords among the preamble codewords deduced by the BS, the preamble codeword utilization can be calculated using Equations (7) and (10) as follows:

(11) $$E=\frac{E[N_{codeword}]}{E[N_{de}]}=\frac{\left(1-{\left(1-\frac{1}{MN_{fp}}\right)}^{\nu }\right)}{\left(1-{\left(1-\frac{1}{M}\right)}^{\nu }\right)\left(1-{\left(1-\frac{1}{N_{fp}}\right)}^{\nu }\right)}.$$

#### 4.1.3. PUSCH Timeout RATE Analysis Model

To prevent redundant preamble codeword recording by the BS, the PUSCH waiting time is set as follows:

(12) $$W=\frac{M}{N_{max,fp}},$$

where $N_{max,fp}$ denotes the maximum number of preamble sequences transmitted in the first RA slot. This prevents the transmission of the same preamble codewords, even when $N_{max,fp}$ is fully utilized for every RAO.

Let $N_{de}\left(t\right)$ be the number of deduced preamble sets at time slot *t*, $N_{w}\left(t\right)$ be the cumulative number of waiting devices at time slot *t*, and G(t) be the number of extra preamble codewords that cannot be served owing to the PUSCH limitations at time slot *t*. In the initial time slot 1, the number of waiting devices at time 1 can be calculated as $N_{w}\left(1\right)=max\left(0,N_{de}\left(1\right)-N_{ST}\right)$, where $N_{ST}$ is the allocable PUSCH resource, which has a fixed value of 144 [30]. If $N_{w}\left(1\right)$ is not zero, it means that some of the preamble codewords cannot receive the RAR message at time t=1 and need to receive PUSCH resources within the PUSCH waiting window *W*. In addition, the PUSCH gain at time 1 can be calculated as $G\left(1\right)=max(0,N_{de}\left(1\right)-N_{ST})$; at time slot 2, $N_{w}\left(2\right)$ includes G(1) when extra preamble codewords exist at time slot t=11; therefore, the number of waiting devices at time 1 can be calculated as $N_{w}\left(2\right)=max\left(w,N_{de}\left(2\right)+G\left(1\right)-N_{ST}\right)$. Furthermore, the PUSCH gain at time 2 can be calculated as $G\left(2\right)=max(0,N_{de}\left(t\right)+G\left(1\right)-N_{ST})$. Therefore, the general formula for G(t) can be formulated as follows:

(13) $$G\left(t\right)=max(0,N_{de}\left(t\right)+G\left(t-1\right)-N_{ST}).$$

At time slot t=W where the PUSCH waiting time has expired, $N_{w}\left(t\right)$ includes the number of timeout preamble codewords $N_{to}\left(t\right)$ as follows:

(14) $$N_{to}\left(t\right)=max\left(0,G\left(t-1\right)+N_{de}\left(t\right)-N_{ST}\cdot W\right),$$

where G(t−1) + $N_{de}$ is the device that waits to receive the RAR message at time slot t−1, and $N_{ST}\cdot W$ is the total allocable PUSCH resources between times t−W and *t*.

The number of waiting devices consists of three main components: the number of deduced preamble codewords, number of previous extra preamble codewords, and number of timeout preamble codewords. As the three aforementioned components are already defined in (10), (13), and (14), $N_{w}\left(t\right)$ can be calculated as follows:

(15) $$N_{w}\left(t\right)=max\left(0,N_{de}\left(t\right)+G\left(t-1\right)-N_{ST}-N_{to}\left(t-W\right)\right).$$

The reason that the number of timeout devices at time t−W is reflected in the waiting preamble codewords at time *t*, is that the removal of the corresponding timeout preamble codewords from the waiting preamble codeword occurs after the PUSCH timeout window *W*.

Because the PUSCH timeout rate is the ratio of the number of timeout preamble codewords to the deduced preamble codewords, the PUSCH timeout rate in all time slots *t* can be calculated using (14) and (15) as follows:

(16) $$T=\frac{N_{to}\left(t\right)}{N_{de}\left(t\right)}=\frac{max(0,max\left(0,N_{de}\left(t+W\right)+G\left(t+W\right)-N_{ST}-N_{to}\left(t\right)\right)+N_{de}\left(t\right)-N_{ST}\cdot W)}{M\cdot N_{fp}\left(1-{\left(1-\frac{1}{M}\right)}^{\nu }\right)\left(1-{\left(1-\frac{1}{N_{fp}}\right)}^{\nu }\right)}.$$

#### 4.1.4. The Proposed Preamble Codeword Set Selection Algorithm

The BS performs the preamble codeword set selection algorithm as shown in Algorithm 1. The proposed preamble codeword set selection Algorithm 1 selects the optimal preamble codeword set that maximizes the RA success rate based on the number of active device estimations. In lines 3, 4, and 5 of Algorithm 1, the BS performs the preamble transmission success rate analysis, the preamble codeword utilization rate analysis, and the PUSCH timeout rate analysis for each candidate scale of the preamble codeword sets. In Figure 4, the preamble transmission success rate indicates the success rate up to the preamble transmission in phase 2. Preamble codeword utilization represents the proportion of actual transmission preamble codewords among the inferred preamble codewords. The PUSCH timeout rate indicates the proportion of the number of PUSCH allocations among the successfully transmitted preamble codewords in phase 4. Because all the aforementioned conditions should be satisfied for the entire RA to succeed, the RA success rate can be defined as a product of the three aforementioned ratios and is calculated in line 6. Finally, Algorithm 1 selects and returns the $N_{fp}$ value that maximizes the RA success rate in lines 7, 8, and 9. The BS can calculate the selectable preamble in the RA cycle based on the last preamble index of the first RA slot and $N_{fp}$ value. After the determination of the preamble sequences in the first RA slot, the BS broadcasts the selectable preamble sequences in the first RA slot through the SIB-2 message. Algorithm 1’s computational complexity is $O(N_{fp})$. Since the complexity of the proposed algorithm is low, it is possible for the BS to perform Algorithm 1 in each RA cycle. Algorithm 1 is performed in an online manner and the optimal preamble codeword set is selected based on the number of active device estimation values in every RA cycle. This means that the proposed CeRA-eSP technique can operate efficiently without complex computing operations and state information collection phases.
**Algorithm 1** Preamble codeword set selection in the base station**Input:**ν**Output:**$N_{fp}$1:**for**i=2 to $N_{fp,max}$ **do**2:   $R_{max}\leftarrow 0$3:   $S=\frac{\nu e^{-\frac{\nu }{M\cdot i}}}{M\cdot i}$4:   $E=\frac{M\cdot i\left(1-{\left(1-\frac{1}{M\cdot i}\right)}^{\nu }\right)}{M\cdot i\left(1-{\left(1-\frac{1}{i}\right)}^{\nu }\right)\left(1-{\left(1-\frac{1}{i}\right)}^{\nu }\right)}$5:   $T=\frac{N_{to}\left(t\right)}{N_{de}\left(t\right)}=\frac{max(0,max\left(0,N_{de}\left(t+W\right)+G\left(t+W\right)-N_{ST}-N_{to}\left(t\right)\right)+N_{de}\left(t\right)-N_{ST}\cdot W)}{M\cdot N_{fp}\left(1-{\left(1-\frac{1}{M}\right)}^{\nu }\right)\left(1-{\left(1-\frac{1}{N_{fp}}\right)}^{\nu }\right)}$6:   R=S·E·T7:   **if** ($R_{max}<R$) **then**8:       $R_{max}\leftarrow R$9:       $N_{fp}\leftarrow i$10:   **end if**11:**end for**

### 4.2. Improved PUSCH Allocation Scheme

**Figure 5:**
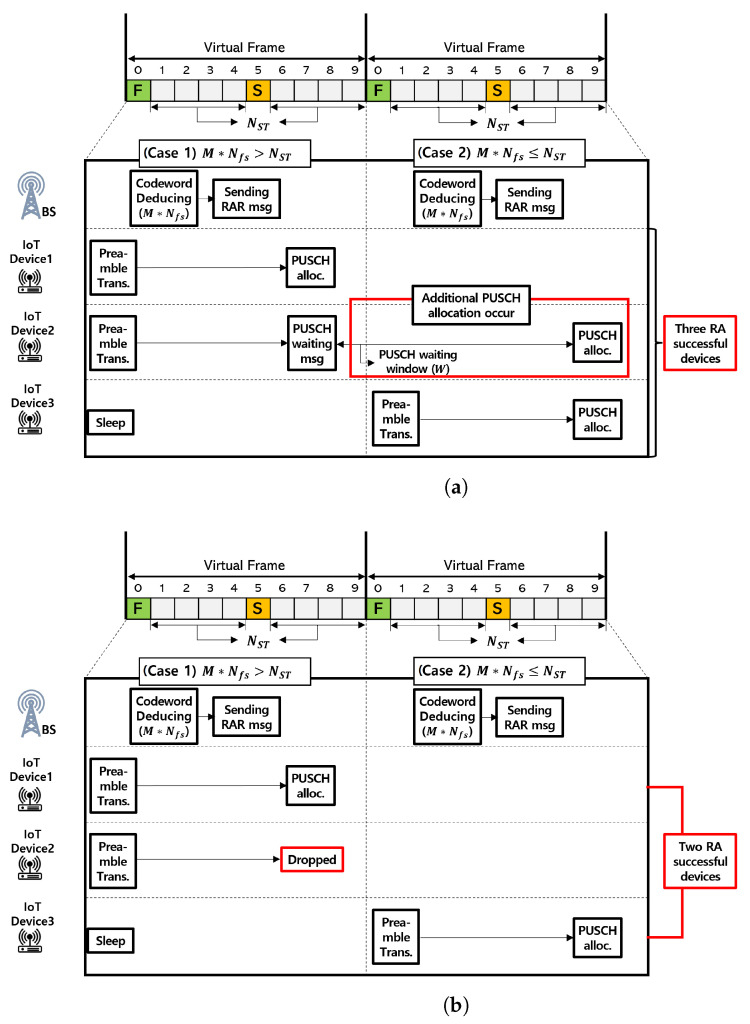
PUSCH allocation scheme comparison. (**a**) Legacy PUSCH allocation scheme. (**b**) Improved PUSCH allocation scheme.

We propose an improved PUSCH allocation scheme to mitigate the degradation of PUSCH resources. To improve the utilization of PUSCH resources, we extend the waiting message proposed in [31] to the PUSCH waiting message as shown in Figure 5. The traditional RA method drops devices when PUSCH resources are insufficient, even though a successful preamble transmission has already been achieved. In Figure 5, the legacy PUSCH allocation drops the second device when the number of deduced preamble codeword sets is larger than the allocable PUSCH resources in one RA cycle. By contrast, the improved PUSCH allocation procedure first transmits a PUSCH waiting message to devices that have successfully transmitted the preamble, allowing devices to wait for a certain waiting window time without dropping. Because the access intensity is different for each RAO, the remaining PUSCH resources at low access intensity may be allocated to devices waiting for the RAR message. This means that the utilization of PUSCH resources is increased, thereby alleviating the PUSCH resource utilization degradation problem caused by large-scale preamble codewords.

In the proposed PUSCH allocation scheme, the BS temporarily records a successfully transmitted preamble codeword. Because the BS cannot obtain specific information from the device that transmits the preamble codeword, PUSCH resources are allocated to the preamble codeword based on the first-come first-out method [16,24,32]. If the RAR message is received within the PUSCH waiting time, the device may proceed to the connection-request process. Otherwise, the device performs an RA reattempt because the PUSCH waiting time has expired.

## 5. Performance Evaluation

### 5.1. Simulation Environment

We developed a simulator in Python 3.7 to measure the performance of the proposed CeRA-eSP scheme. Table 1 lists the system parameters used in the simulations. We implemented an mMTC scenario with N=100,000 devices in single-cell access during 4000 RAO. In the case of the CeRA method, an experiment was performed in an environment with a codeword length $L_{c}=2$ and a situation in which devices were connected for a total of 2000 virtual frames. Each experiment was performed 100 times, and the average value of the experimental results was used as the measurement value.

**Figure 6:**
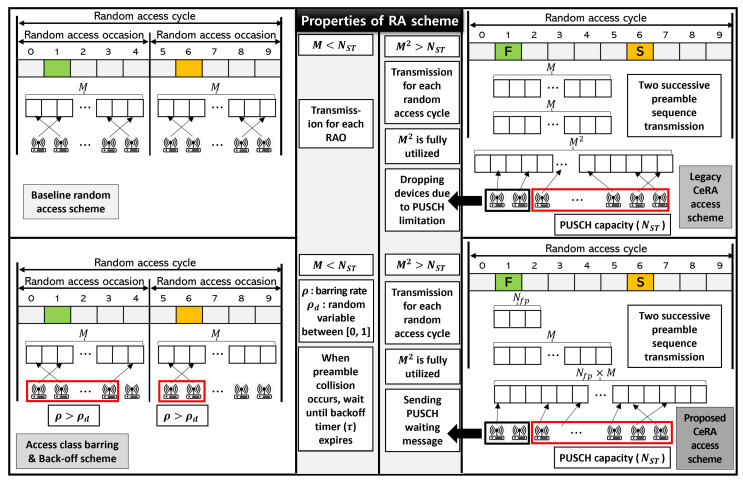
Comparison of different random access schemes.

We compared the performance of the proposed CeRA-eSP scheme with legacy schemes such as baseline, access class barring, back-off, and legacy CeRA. The operation summary of each random access method is described below and Figure 6 illustrates the comparison of the four different random access schemes.

- Baseline: This refers to the existing RA method in which each device transmits a random preamble sequence from a set of 54 contention-based preambles. Because congestion control is not considered, activated devices transmit a preamble on every RA occasion. Moreover, because the number of total preamble sequences is less than the number of available PUSCH resources, a successful preamble transmission means that PUSCH resource allocation is also successful.
- Access class barring and back-off (ACB and BO): This refers to the proposed ACB and BO technique for solving the high access intensity problem in the mMTC scenario [11,12,13]. Activated devices select an arbitrary value between zero and one. When the value is less than the access barring rate, the device is barred for a certain duration. In terms of the preamble transmission, the devices transmit a random preamble sequence among 54 contention-based preambles, similar to the baseline scheme. When a preamble collision occurs, the device waits for the random back-off time between zero and the given back-off time duration and then retransmits the preamble sequence again. Because the number of preamble sequences in the ACB and BO scheme is less than the number of available PUSCH resources, the restriction of PUSCH resources is not considered in the baseline.
- Legacy CeRA: This refers to an existing CeRA transmission scheme in which PUSCH resource constraints are not considered. Because the codeword length of the target system is 2, devices select one of the $54^{2}=2916$ preamble codewords in the preamble transmission step. Because congestion control is not considered, activated devices transmit preamble codewords in every RA cycle. CeRA utilizes two consecutive RAOs as one RA cycle; thus, the number of RA cycles considered in the simulation is 2000. If the number of PUSCH resources is less than the number of deduced preamble codewords, the BS drops the extra preamble codewords.
- The proposed CeRA-eSP: In the same way as legacy CeRA, one of the preamble codewords is randomly selected to perform the preamble transmission. The main difference between the proposed CeRA-eSP method and the legacy CeRA method is that a different scale of preamble codeword sets can be selected according to the access intensity. If the number of available PUSCH resources is less than the deduced number of preamble codewords, the BS sends a PUSCH waiting message to the extra devices. The proposed CeRA-eSP method transmits a preamble codeword in 2000 RA cycles in the same way as legacy CeRA.

### 5.2. Simulation Results

#### 5.2.1. Delay and RA Failure Rate

**Figure 7:**
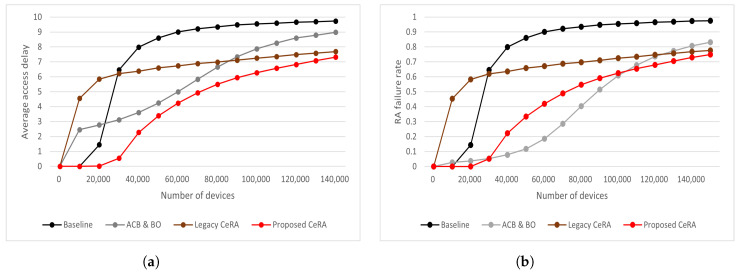
(**a**) Average delay for different consistency. numbers of devices and (**b**) RA failure rate for different numbers of devices.

In Figure 7, the average delay and RA failure rate for the RA schemes for different numbers of devices are analyzed. The average time of the total RA procedure was measured to analyze the average delay for the RA schemes, including devices that failed RA, as shown in Figure 7a. In the baseline method, most devices were dropped as the number of access devices increased to over N=20,000. As the number of dropped devices increased, the average access delay of the baseline method increased significantly, even at low access intensities. The ACB and BO method showed a lower access delay performance than the legacy CeRA method in a low-access-intensity environment. Because additional access delay occurs owing to the back-off time, it shows a larger access delay increasing tendency than legacy CeRA as the number of devices increases. Furthermore, as the number of access devices exceeds N=120,000, the ACB and BO method shows a higher RA failure rate than legacy CeRA. Consequently, the average access delay exceeds that of legacy CeRA.

In Figure 7b, it can be seen that the proposed CeRA-eSP method exhibited a lower RA failure rate than the legacy CeRA method in all situations. Compared with the legacy CeRA method, the proposed CeRA-eSP method achieved a lower RA failure rate by dynamically selecting the preamble codeword set. As the access intensity increased, the proposed CeRA-eSP method utilized a higher preamble codeword set. In a situation where the number of access devices was N=150,000, the proposed CeRA-eSP method utilized a larger preamble codeword than the legacy CeRA method; therefore, the RA failure rates of the two methods were similar. All the RA schemes showed an approximately linear increase in the number of dropped devices. The baseline method showed the highest RA failure rate. The baseline dropped most of the devices because the high collision problem occurred in the process of supporting large access intensity with limited preamble sequences that lacked a baseline and had no congestion control. The ACB and BO method showed a low RA failure rate when the number of devices was less than N=50,000. However, the ACB and BO method had the highest rate of increase in the RA failure rate as the access intensity increased. This is because the ACB and BO method did not efficiently support high access intensity with limited preamble resources as the baseline method did. In addition, the baseline and ACB and BO methods showed similar performances as the number of devices increased.

#### 5.2.2. Success Rates of Preamble Transmission and Random Access

**Figure 8:**
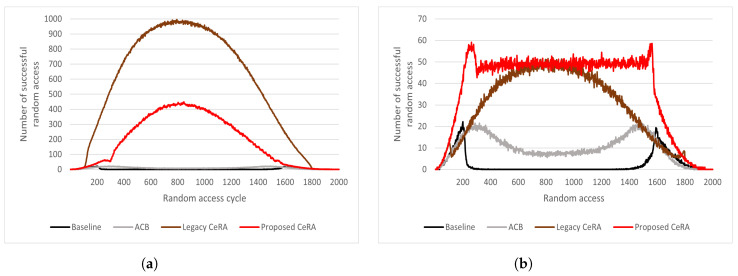
(**a**) Number of Successful preamble transmissions and (**b**) Number of successful random accesses.

Figure 8 shows the RA performance during the 2000 RA cycle. In Figure 8a, it can be seen that the baseline and ACB and BO schemes showed a nearly zero preamble transmission rate in high access intensity. Due to the preamble codeword expansion, the CeRA schemes showed outstanding performance in respect of the preamble transmission. The legacy CeRA method showed a larger number of successful preamble transmissions than the proposed CeRA-eSP method owing to the low preamble collision rate of the legacy CeRA method. This is because legacy CeRA utilizes 2916 preamble codeword sets without considering preamble codeword utilization. Despite the number of successful preamble transmissions being larger than the CeRA-eSP method, the BS could not allocate PUSCH resources to all successful preamble codewords owing to PUSCH resource limitations. Therefore, Figure 8b shows that the proposed CeRA-eSP method achieved a larger number of successful RAs than the legacy CeRA method. Due to the proposed CeRA-eSP method selecting the optimal preamble codeword set by considering PUSCH resource limitations, a higher number of successful RAs can be achieved. In the case of the baseline method and the ACB and BO method, the simulation results showed that the number of successful RAs was lower than the CeRA schemes in high-access-intensity situations as the preamble transmission rate was low.

In both the legacy CeRA and proposed CeRA-eSP methods, fluctuations occurred at certain points as the access intensity increased. The fluctuation point occurred when the penalty owing to a lack of PUSCH resources was reversed, and the gain owing to a decrease in the preamble collision rate of the CeRA was reversed. Because both schemes have the same PUSCH resource limit, the performance curve after the fluctuation at RAO=300 showed a similar shape. This fluctuation occurred when the preamble codeword transmitted from one RAO exceeded the allocable PUSCH resources. Because both schemes had the same PUSCH resource limit, the performance curve after the fluctuation showed a similar tendency.

#### 5.2.3. Trade-Off Analysis between Preamble Transmission Rate and Random Access Rate

**Figure 9:**
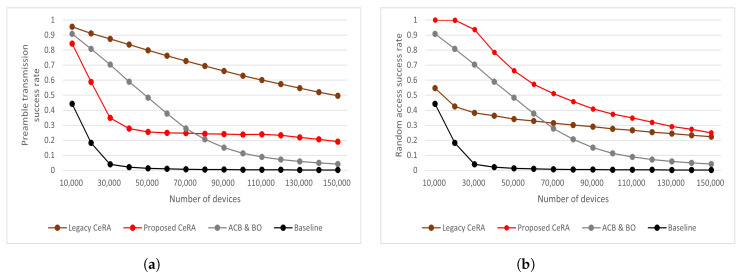
(**a**) Successful preamble transmission rate for different numbers of devices and (**b**) Successful random access rate for different numbers of devices.

Figure 9 shows the RA step performance for the various RA schemes. The baseline and ACB and BO schemes utilized fewer preamble sequences than allocable PUSCH resources; therefore, the preamble transmission success rate and RA success rate had the same value as mentioned previously. As shown in Figure 9a, the legacy CeRA method achieved the highest preamble transmission success rate. This is because legacy CeRA utilized the largest preamble codeword set. The ACB and BO method showed a higher preamble transmission success rate than the proposed CeRA-eSP method when the number of devices was less than N=70,000. This is because the ACB and BO method maintained the number of access devices in one RAO by utilizing the barring rate. As the access intensity increased, the ACB and BO scheme converged to a preamble transmission success rate similar to the baseline because of the limited number of preamble sequences. The proposed CeRA-eSP method utilized the preamble codeword, which is an expanded form of the preamble sequence; thus, it showed a higher preamble transmission success rate than the ACB and BO method in high-access-intensity situations.

In Figure 9b, it can be seen that the proposed CeRA-eSP method achieved the highest RA success rate in all cases. The baseline and ACB and BO methods degraded RA performance owing to the high collision problem. The legacy CeRA method degraded RA performance owing to the codeword ambiguity problem, which was caused by a large preamble codeword set of 2916. The proposed CeRA-eSP method achieved the highest RA success rate by considering the preamble collision, codeword utilization, and PUSCH timeout rates. Therefore, the legacy CeRA scheme consumed PUSCH resources in a phantom codeword and achieved less than a 60% RA success rate, whereas the proposed CeRA-eSP method achieved the highest RA success rate. Even in the high-access-intensity situation of N=150,000, the proposed CeRA-eSP method achieved an RA success rate of 25%.

#### 5.2.4. Comparison of RA Success Rate of the Proposed CeRA-eSP and Legacy CeRA Methods

**Figure 10:**
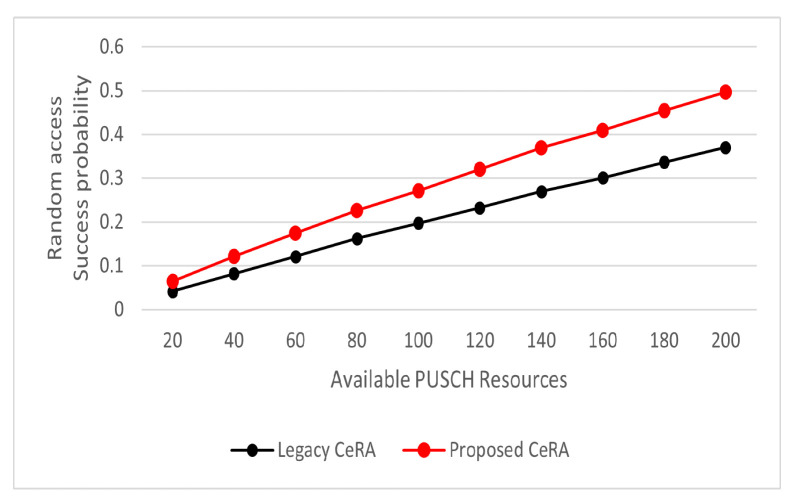
RA success rates for different available PUSCH resources.

According to the previous performance analysis results, it can be observed that the legacy CeRA method was significantly affected by PUSCH resources. Because a specific PUSCH resource could have had a significant impact on the legacy CeRA and the proposed CeRA-eSP methods, additional simulations were performed to analyze the RA success rates according to the PUSCH resources. In Figure 10, it can be observed that the RA success rate increased linearly according to the number of resources for both the legacy CeRA and the proposed CeRA-eSP methods. The proposed CeRA-eSP method achieved a higher RA success rate than the legacy CeRA scheme for all the PUSCH resources considered. This is because the proposed CeRA-eSP method utilized the PUSCH waiting message to increase the utilization of the PUSCH resources. Because the utilization of PUSCH resources was high, it can be observed that the performance of the proposed CeRA-eSP method was higher even with the operation of additional PUSCH resources.

#### 5.2.5. PUSCH Wastage of Legacy CeRA and CeRA-eSP

**Figure 11:**
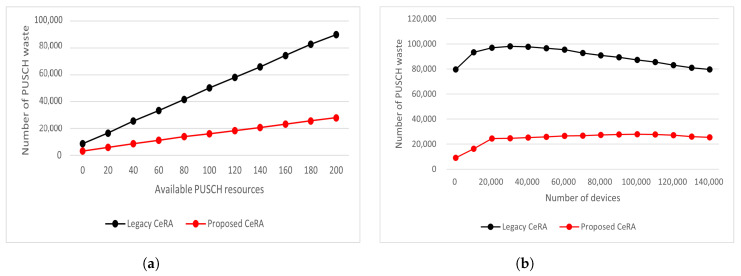
(**a**) PUSCH wastage for different PUSCH resources and (**b**) PUSCH wastage for different numbers of devices.

In Figure 11, the PUSCH wastage of legacy CeRA and CeRA-eSP for various PUSCH resources and device densities is shown. In Figure 11a, it can be seen that CeRA-eSP generated less PUSCH waste than legacy CeRA in all cases when N=100,000. Legacy CeRA inferred more phantom codewords due to high codeword ambiguity with the growth of allocable PUSCH resources. In other words, the BS in legacy CeRA had a larger deducable preamble set than that of CeRA-eSP, so relatively more phantom codewords were generated, which resulted in a waste of PUSCH resources. Figure 11b shows that CeRA-eSP achieved low PUSCH wastage in all cases. In legacy CeRA, as the number of devices increased, the number of deduced phantom codewords increased, and as the number of cases in which PUSCH resources were allocated to phantom codewords increased, it was confirmed that up to a 100,000 PUSCH wastage was generated during the entire RA. On the other hand, since the proposed CeRA-eSP method dynamically adjusted the preamble codeword set according to the number of active device estimations, it maintained a low number of phantom codewords and as a result, achieved a low PUSCH wastage performance. It is worth noting that CeRA-eSP achieved high PUSCH efficiency in a highly densified network, which consisted of more than N=120,000 devices. This means that CeRA-eSP saved the precious uplink resources of mMTC by improving the efficiency of resources consumed by RA.

## 6. Discussion

In this study, we proposed a code-expanded RA scheme that considers preamble codeword utilization and PUSCH resource limitations. Most conventional CeRA schemes focus more on the preamble transmission success rate than on the overall RA success rate. To improve the RA success rate, we formalized the analysis model of the preamble transmission success rate, preamble codeword utilization, and PUSCH timeout rate. In addition, we proposed the first RA slot selection procedure and PUSCH waiting message to improve PUSCH resource utilization. In the performance evaluation section, we validated that the proposed CeRA-eSP exceeded the performance of the existing CeRA scheme with respect to the RA success rate. In future work, we will further improve the preamble selection process using machine learning techniques that have been considered in recent studies [19,20,21].

## Figures and Tables

**Table 1:** Simulation Parameters.

Parameters	Values
Traffic distribution model	Beta distribution(α = 3, β = 4)
Time duration	10 (s)
PRACH configuration	6 [21]
Total random access occasion	4000 (n)
Preamble codeword length ($L_{c}$)	2
Available preamble sequence (*M*)	54 (n)
Available PUSCH resources ($N_{ST}$)	144 (n) [26]
Maximum number of $N_{fp}$	27 (n)
Minimum number of $N_{fp}$	2 (n)
Waiting window time (*W*)	10 (ms)
Delay constraint	10 (s)
Maximum number of preamble retransmission	10 (n)
Back off time	20 (ms)
Barring rate	0.3
Mean barring time	1 (s)

## Abbreviations

The following abbreviations have been used in this manuscript:

ACB	access class barring
BS	base station
CeRA	code-expanded random access
mMTC	massive machine-type communications
NOMA	non-orthogonal multiple access
HTC	human-type communication
IoT	Internet of things
ITU-R	international telecommunication union radiocommunication sector
PBCH	physical broadcasting channel
PDCCH	physical downlink control channel
PRACH	physical random access channel
PUSCH	physical uplink shared channel
RA	random access
RACH	random access channel
RAR	random access response
RAO	random access occasion
RL	reinforcement learning
SIB-2	system information block-2
**Notation**	**Description**
*M*	Preamble sequences in one random access slot
$M_{c}$	Number of preamble codewords
$L_{c}$	Preamble codeword length
*W*	PUSCH timeout window size
ν	Mean value of activated devices
*I*	Number of unselected preambles
S	Success probability of preamble transmission
*N*	Number of devices
$N_{fp}$	Number of preambles that are selectable at the first random access slot
$N_{fp,max}$	Maximum number of preambles that can be transmitted at the first random access slot
$N_{sp}$	Number of preambles that are selectable at the second random access slot
$N_{codeword}$	Number of codewords that are transmitted at the random access cycle
$N_{chosen}$	Number of preambles that are transmitted at the first random access slot
$N_{de}$	Number of deduced preambles
$N_{de}\left(t\right)$	Number of deduced preambles at time slot *t*
$N_{ST}$	Number of allocable PUSCH resources for each device
$N_{to}\left(t\right)$	Number of devices that are in the state of PUSCH timeout at time slot *t*
$N_{w}\left(t\right)$	Number of cumulative waiting devices at time slot *t*
G(t)	Number of extra preamble codewords that cannot be served owing to a PUSCH limitation at time slot *t*
$P_{chosen}$	Probability that a specific preamble codeword is chosen by at least one device

## References

[1] Navarro-Ortiz J., Romero-Diaz P., Sendra S., Ameigeiras P., Ramos-Munoz J.J., Lopez-Soler J.M. (2020). A survey on 5G usage scenarios and traffic models. IEEE Commun. Surv. Tutor..

[2] Shayea I., Ergen M., Azmi M.H., Çolak S.A., Nordin R., Daradkeh Y.I. (2020). Key challenges, drivers and solutions for mobility management in 5G networks: A survey. IEEE Access.

[3] Guo S., Lu B., Wen M., Dang S., Saeed N. (2022). Customized 5G and Beyond Private Networks with Integrated URLLC, eMBB, mMTC, and Positioning for Industrial Verticals. IEEE Commun. Stand. Mag..

[4] ITU-R (2017). Minimum Requirements Related to Technical Performance for IMT–2020 Radio Interface(s).

[5] 3GPP (2017). Study on New Radio (NR) Access Technology Physical Layer Aspects.

[6] Shafi M., Molisch A.F., Smith P.J., Haustein T., Zhu P., Silva P., Wunder G. (2017). 5G: A tutorial overview of standards, trials, challenges, deployment, and practice. IEEE J. Sel. Areas Commun..

[7] Sharma S.K., Wang X. (2019). Toward massive machine type communications in ultra-dense cellular IoT networks: Current issues and machine learning-assisted solutions. IEEE Commun. Surv. Tutor..

[8] Salam T., Rehman W.U., Tao X. (2019). Data aggregation in massive machine type communication: Challenges and solutions. IEEE Access.

[9] Vural S., Wang N., Foster G., Tafazolli R. (2018). Success probability of multiple-preamble-based single-attempt random access to mobile networks. IEEE Commun. Lett..

[10] Pratas N.K., Thomsen H., Stefanović Č., Popovski P. (2012). Code-expanded random access for machine-type communications. IEEE Globecom Work..

[11] Liu Y., Deng Y., Jiang N., Elkashlan M., Nallanathan A. (2020). Analysis of random access in NB-IoT networks with three coverage enhancement groups: A stochastic geometry approach. IEEE Trans. Wirel. Commun..

[12] Jiang N., Deng Y., Nallanathan A., Kang X., Quek T.Q. (2018). Analyzing random access collisions in massive IoT networks. IEEE Trans. Wirel. Commun..

[13] Leyva-Mayorga I., Rodriguez-Hernandez M.A., Pla V., Martinez-Bauset J., Tello-Oquendo L. (2019). Adaptive access class barring for efficient mMTC. Comput. Netw..

[14] Liu W., Cui Y., Ding L., Sun J., Liu Y., Li Y., Zhang L. Joint Optimization of Preamble Selection and Access Barring for MTC with Correlated Device Activities. Proceedings of the IEEE International Conference on Communications Workshops (ICC Workshops).

[15] Bui A.T., Nguyen C.T., Thang T.C., Pham A.T. (2019). A Comprehensive Distributed Queue-Based Random Access Framework for mMTC in LTE/LTE-A Networks with Mixed-Type Traffic. IEEE Trans. Veh. Technol..

[16] Kim T., Jang H.S., Bang I., Ko K.S. (2021). Access Priority Provisioning Based on Random Access Parallelization for Prioritized Cellular IoT. IEEE Access.

[17] Li Z., Wang Y., Wang T., Wang Z. Joint Access Control and Resource Allocation for mMTC based on Tagged Preamble. Proceedings of the International Conference on Wireless Communications and Signal Processing (WCSP).

[18] Bockelmann C., Pratas N.K., Wunder G., Saur S., Navarro M., Gregoratti D., Dekorsy A. (2018). Towards Massive Connectivity Support for Scalable mMTC Communications in 5G Networks. IEEE Access.

[19] Abera W., Olwal T., Marye Y., Abebe A. Learning Based Access Class Barring for Massive Machine Type Communication Random Access Congestion Control in LTE-A Networks. Proceedings of the International Conference on Electrical, Computer and Energy Technologies (ICECET).

[20] Bui A.T., Pham A.T. (2020). Deep Reinforcement Learning-Based Access Class Barring for Energy-Efficient mMTC Random Access in LTE Networks. IEEE Access.

[21] Bai J., Song H., Yi Y., Liu L. (2021). Multiagent Reinforcement Learning Meets Random Access in Massive Cellular Internet of Things. IEEE Internet Things.

[22] Miuccio L., Panno D., Riolo S. (2021). A New Contention-Based PUSCH Resource Allocation in 5G NR for mMTC Scenarios. IEEE Commun. Lett..

[23] Bai Y., Chen W., Ai B., Zhong Z. Contention Based Massive Access Scheme for B5G: A Compressive Sensing Method. Proceedings of the International Wireless Communications and Mobile Computing (IWCMC).

[24] Ye N., Wang A., Li X., Yu H., Li A., Jiang H. (2017). NOMA-Based Random Access with Multichannel ALOHA. IEEE J. Sel. Areas Commun..

[25] Astudillo C.A., Hossain E., Fonseca N.L. (2021). Random Access Based on Maximum Average Distance Code for Massive MTC in Cellular IoT Networks. IEEE Wirel. Commun. Lett..

[26] Jiang H., Qu D., Ding J., Jiang T. (2019). Multiple Preambles for High Success Rate of Grant-Free Random Access with Massive MIMO. IEEE Trans. Wirel. Comminications.

[27] 3GPP (2011). Study on RAN Improvements for Machine Type Communications.

[28] Vural S., Wang N., Foster G., Tafazolli R. Online control of preamble groups with priority in cellular IoT networks. Proceedings of the IEEE INFOCOM.

[29] Haas J., Deng J. (2003). On optimizing the backoff interval for random access schemes. IEEE Trans. Commun..

[30] Miuccio L., Panno D., Riolo S. (2020). Joint control of random access and dynamic uplink resource dimensioning for massive MTC in 5G NR based on SCMA. IEEE Internet Things J..

[31] Lee B.H., Lee H.S., Moon S., Lee J.W. (2020). Enhanced Random Access for massive-Machine-Type Communications. IEEE Internet Things J..

[32] Fayaz M., Yi W., Liu Y., Nallanathan A. (2021). Transmit power pool design for grant-free NOMA-IoT networks via deep reinforcement learning. IEEE Trans. Wirel. Commun..

